# Alasdair MacIntyre on the division of goods and “the corrupting power of institutions”

**DOI:** 10.3389/fsoc.2022.986184

**Published:** 2022-11-10

**Authors:** Tony Burns

**Affiliations:** School of Politics and International Relations, University of Nottingham, Nottingham, United Kingdom

**Keywords:** MacIntyre, social institutions, Aristotle, Plato, division of goods

## Abstract

This paper examines the distinction between “internal goods” and “external goods” and its significance for the political thought of Alasdair MacIntyre, focusing especially on its relevance for our understanding of MacIntyre's views regarding the relationship which exists between “practices” and social “institutions. ” The paper explores the origins of this distinction in the writings of Plato and Aristotle, both of whom (like MacIntyre) associate the notion of external goods with such things as wealth, status and power. Plato argues that these things are not really “goods” at all, but rather “bads,” or things which ought to be avoided. Aristotle, on the other hand, takes issue with that view, arguing that the pursuit of such things is acceptable, morally speaking, provided it is in moderation and not to excess. The paper argues that what MacIntyre says about external goods and “the corrupting power of institutions” in *After Virtue* is ambivalent. For this reason, his views are open to different possible interpretations. Most commentators have read and understood him as a follower of Aristotle. There is however a strain of Platonism at times in the critical remarks which he makes about social institutions and those who manage them.

“[T]he essential function of the virtues is clear. Without them, without justice, courage and truthfulness, practices could not resist the corrupting power of institutions.”

Alasdair MacIntyre

## Introduction

Alasdair MacIntyre has a long-standing interest in social institutions and their management. This can be found in a number of his writings before the publication of *After Virtue: A Study in Moral Theory* in 1981. It is central to that work. It can also be found in his most recent major publication, *Ethics in the Conflicts of Modernity*, which was published in 2016 (MacIntyre, 2016). In this article I focus attention on what I shall take to be the classical statement of MacIntyre's views on this subject in *After Virtue*, especially on his opinion, expressed in the well-known and often cited quotation above, that social institutions possess a corrupting power. I do not consider whether MacIntyre continues to hold this view in his more recent writings.

Although quite a lot has been written about MacIntyre's views regarding practices and social institutions, and the relationship which exists between them, much less have been written about what MacIntyre has to say about “goods,” about the different types of “good” which he thinks there are, and how the distinction which exists between these types of good maps on to his distinction between practices and institutions (Höpfl and Beadle, 2003; Knight, 2008, 2011). This omission is surprising, given that in *After Virtue* MacIntyre defines the concept of a “practice,” as he understands it, by reference to a particular type of “good.” For example, he says there that “by a ‘practice' I am going to mean any coherent and complex form of socially established co-operative human activity through which goods internal to that form of activity are realized” (MacIntyre, 2007 [1981], p. 187). He also maintains that there are “two kinds of goods,” and that in addition to the internal goods of practices, there is also a second category of good, namely “external goods,” which includes “those goods” which are “externally and contingently attached” to social practices “by the accidents of social circumstance.” For example, “such goods as prestige, status and money” (MacIntyre, 2007 [1981], p. 188). Moreover, MacIntyre also defines what is for him the all important concept of “virtue” by reference to his distinction between different types of good. In *After Virtue* he states that “a virtue is an acquired human quality the possession and exercise of which tends to enable us to achieve those goods which are internal to practices and the lack of which effectively prevents us from achieving any such goods” (MacIntyre, 2007 [1981], p. 191). Given these remarks, it seems to me that MacIntyre's distinction between the different types of good, especially internal goods and external goods, merits closer attention than it has received hitherto.

The origin of MacIntyre's distinction between the different types of good is to be found in the philosophy of the ancient Greeks, especially in the writings of Plato and Aristotle, both of whom focus their attention on the issue of what is “good” for individual moral agents or persons, all of whom are assumed to be human beings (Aristotle, 1984; Plato, 1997a). MacIntyre is commonly regarded as a follower of Aristotle rather than of Plato. Indeed, as shown below, whenever he discusses Plato and his ideas he has a tendency to criticize them from a point of view that is recognizably Aristotelian. Despite this, however, it seems to me that MacIntyre is not entirely consistent when discussing this issue and that there is an undertone of Platonism in the remarks which he makes at times about contemporary social institutions and their managers. This is true especially when in *After Virtue* he talks about “the corrupting power of institutions” (MacIntyre, 2007 [1981], p. 194). This is a highly significant remark which is invariably referred to by those commenting on MacIntyre's ideas and often repeated, in my view uncritically, without any discussion of the underlying assumptions upon which it is based. The point of the present article is to draw out what those assumptions are. The article has two parts. In the first, I will say something about the Greeks, specifically the debate between Plato and Aristotle around the concept of the good and the classification of the different types of goods. In the second, I turn to consider what MacIntyre has to say about this same issue, connecting his views on the subject to those of Plato and Aristotle. I also consider the relevance of this for our assessment of the significance of his well-known reference to “the corrupting power of institutions” in *After Virtue*.

## Plato and Aristotle on the division goods

### Plato on the division of goods

Plato says different things at different times about the division of “goods.” What follows, therefore, is a selective reading of his views, bearing in mind what MacIntyre has to say about them. According to one reading of his views, Plato argues that the goods which are associated with the human body and its pleasures or desires are not really genuine goods at all. On the contrary, they are “bads.” As such, as Tom Angier has pointed out, they are “necessarily undesirable” (Angier, 2020, p. 101). That is to say, they are ethically undesirable, even if is true that some people do in fact desire them. Plato holds that they ought as a matter of principle to be avoided. He also holds that those who think that they are really and truly goods, and that they can and do contribute to human happiness, properly understood, are in error. Hence, of course, according to Plato, it is possible in principle, and sometimes (or even often) happens in practice, that one or more individuals, perhaps even an entire society and its laws, can be mistaken when it comes to the question of what is really and truly good for them. Plato regards this as being in part a cognitive error, but one which has moral or ethical consequences. It is, therefore, a moral failing as well as an intellectual one.

This is, perhaps, the view which is most commonly associated with Plato. Nor is this surprising, as there is ample evidence to support it. For example, according to Socrates in *Philebus*, the idea that bodily pleasure, or “animal passion,” is the highest good for human beings is erroneous, even though “all the cattle and horses and the rest of the animals gave testimony” to it (Plato, 1997b, p. 66e−67b, 456). Similarly, in the *Meno*, Plato appears to agree with the view expressed by Aristotle in Book I of the *Nicomachean Ethics*, that “the good is that at which all things aim.” In Plato's case, however, this idea is associated with the view that it is possible for individual agents to be mistaken in their own assessment of what is really or truly “good” for them, and productive of their own happiness, properly understood.

In Plato's dialogue, the character Meno asserts at one point that at least some men (sic) desire “bad things.” This view is rejected by Socrates, who argues that “those who do not know things to be bad” do not in fact “desire what is bad.” Rather, they erroneously “desire those things that they believe to be good but that are in fact bad.” From which it follows, Socrates points out, that “those who have no knowledge of these things and believe them to be good clearly desire good things” and not bad ones (Plato, 1997c, p. 77b, 877). Plato states that the things in question, which are commonly though wrongly thought to be goods, include wealth, status and power. He suggests on numerous occasions that these things are not really goods at all. On the contrary, they are bad, that is, things which ought to be avoided. To think of them as good is to make a mistake.

In Book II of the *Republic*, Plato distinguishes between three different categories of “good” (Plato, 1997a, p. 357a1–d2, 998–999). One of these includes those things which either are or ought to be valued as ends in themselves, or for their “own sake” (Plato, 1997a, p. 357b5–7, 998). A second category includes those instrumental goods which are a necessary means for the achievement of ends which are of value for their own sake. These are things, he says, which “we wouldn't choose for their own sakes, but rather for the sake of” what “comes from them” (Plato, 1997a, p. 357ac4-d1, 999). Plato's third category includes those “goods” which, he says, are of value both for their own sake and also “for the sake of what comes from them” (Plato, 1997a, p. 357c1-2, 999). His idea here is that although the goods in this third category are not instrumental goods, nevertheless their successful pursuit does seem to make a significant contribution to human happiness. This is an accidental or unintended consequence of the successful pursuit of this third type of good.

One of the points at issue in the debate between Socrates and the other characters in the dialogue, especially Thrasymachus, has to do with where justice should be placed within this tri-partite theoretical schema. Should it be placed in the second of the above categories or in the third? Socrates asserts that it is commonly held that justice should be placed in the second category (Plato, 1997a, p. 358a4-8, 999). On that view, he argues, living a life of justice is something which is not to be valued for its own sake. Rather, it considered to be of instrumental value only. It is “practiced for the sake of the rewards and popularity” that come with it. For these are the things which are usually associated (wrongly in Plato's view) with human happiness. Against this commonly held view, Plato argues that justice actually falls into the third category of “good” identified above and not the second. As Socrates puts it, “justice is one of the greatest goods, the ones that are worth getting for the sake of what comes from them,” such as wealth and reputation, “but much more so for their own sake” (Plato, 1997a, p. 367c, 1007). He also maintains that it is for this reason only that a life devoted to justice really and truly “benefits its possessors” (Plato, 1997a, p. 367d, 1007).

According to Plato in the *Republic*, those who hold the commonly held view of justice are mistaken about what it is that happiness, properly understood, actually involves. For they erroneously believe that happiness has to do only with the goods of the body and those external goods such as wealth and reputation with which the goods of the body are connected. Against that view, Plato associates happiness properly understood with the life of the mind or soul. This involves purely intellectual pursuits, such as those having to do with philosophy. However, more practically, such a life is also an ethical life, or a life that is devoted to the cultivation of the virtues, including the virtue of justice. Plato argues that although such a life is or ought to be valued for its own sake, rather than instrumentally, nevertheless one of the consequences of living such a life is that the person who does so will be truly happy, as he understands the term. Moreover, he also claims that living a life of justice may have the further consequence of bringing with it those material things such as status and wealth which are wrongly thought by some to be goods because they are mistakenly thought to make a contribution to human happiness.

Later on in the *Republic*, in Book VI, Socrates again refers to certain “so-called goods, such as wealth and other similar advantages” (Plato, 1997d, p. 495a6-7, 1117). It is, he maintains, pursuit of things of this kind, which are erroneously thought to be goods, rather than what they really are, namely bads, which is to be associated with the notion that the “best nature” of human beings has been, or is being, “destroyed and corrupted.” Hence, it might be said that those who recommend or encourage the living of a life devoted to the pursuit of such things “do the greatest evils” both to cities and to their individual citizens (Plato, 1997d, p. 495a11–13, 1117).

Finally, in *The Laws*, Plato has his “Athenian” character assert that because “our aim in life should be goodness and the spiritual virtue appropriate to mankind” (Plato, 1997e, p. 770c7–d1, 1444), it follows that the Cretans should have “no truck with other pursuits which aim at different ‘goods' (as people call them)” (Plato, 1997e, p. 771a3–4, 1444). He also says that the individual identified above would still be wretched and miserable, “even if all his actions and possessions” were associated with the successful pursuit of those things which “people commonly call “good” (Plato, 1997e, p. 660e4-661a1, 1351–1352). Finally, he states that “these things men usually call “good,” such as wealth and reputation, are in fact “misnamed” (Plato, 1997e, p. 661a6, 1352). Again, therefore, Plato suggests that these things are not in fact goods at all. Rather, they are intrinsically bad. As such they ought to be avoided on ethical grounds. Nor as a matter of fact, Plato claims, will they make people truly “happy,” as he understands that term. According to Plato, on this reading of his views, which does have evidential support, it is not the fact that these alleged goods are pursued to excess which is a problem. Rather, it is the fact that they are pursued at all. This is commonly thought to be the main point of disagreement between Plato's views regarding which things might properly be said to be goods and those of Aristotle.

### Aristotle on the division of goods

Aristotle disagrees with Plato regarding matters of psychology, specifically on the issue of personality, or the nature of the self. Against Plato, Aristotle argues in his *De Anima*, that an individual persons, that is to say, a human being, is a composite entity (Burns, 2000, p. 5–15). This entity is constituted by two component parts, namely a mind or soul and a body. These component parts complement or mutually support one another. Each of them is necessary and neither on its own is sufficient for the existence of an individual person. From this point of view, it may be said that in all individual persons a particular mind or soul has been embodied. This view underpins Aristotle's criticisms of Plato's ideas relating to the division of goods.

Like Plato, and apparently following him, Aristotle also differentiates between different types of good. In the *Nicomachean Ethics* he points out that “goods have been divided into three classes.” He goes on to state that some goods “are described as external,” whereas others are described “as relating to soul or to body.” Moreover, he continues, “we call those that relate to soul most properly and truly goods” (Aristotle, 1984a, I, section 8, p. 1098b11–13, 1736). Aristotle makes similar remarks in the *Politics*, when he observes that “certainly no one will dispute the propriety of that partition of goods which separates them into three classes, viz. external goods, goods of the body, and goods of the soul” (Aristotle, 1984d, VII, section 1, p. 1323a23–26, 2100). By “external goods,” Aristotle has in mind such things as “wealth, property, power, reputation, and the like” (Aristotle, 1984d, VII, section 1, p. 1323a37–38, 2100). Aristotle contrasts these external goods with other types of good, including goods of the body as well as goods of the soul. He assumes that the point of pursuing external goods is that this enables the achievement of those of the body and the soul.

A common reading of Aristotle's understanding of Plato and his ideas is that he attributes to Plato the view that the “goods” of the body, together with the external goods which produce them, are not really or genuinely goods at all, but bads, or things which ought to be avoided. He then criticizes Plato for holding that view. According to this reading of Aristotle, he distances himself from what he takes to be Plato's view by arguing that such things as wealth, status and power, are not in themselves bad. Nor is the pursuit of them necessarily something which ought morally speaking to be avoided, in any and all circumstances. On the contrary, pursuit of these things is permissible, even desirable, up to a point, because it is necessary to satisfy basic (natural or biological) human needs.

In his *Nicomachean Ethics*, Aristotle discusses the relationship which exists between the concept of happiness and that of pleasure. There he does not say that the pursuit of pleasure is in itself a bad thing, even in the case of those pleasures which are associated with the human body and its desires. For, he argues, there are different kinds of pleasure, only some of which are bad. Moreover, in the case of some pleasures at least, including some bodily pleasures, one's assessment of whether their pursuit, in any given case, is a good or a bad thing, ethically speaking, is a matter of degree. For it is possible to pursue pleasures of that kind either in moderation or to excess.

These qualifications notwithstanding, Aristotle maintains that in general the pursuit of pleasure may legitimately be regarded as being “necessarily a good.” Hence the external things which make at least some pleasures possible should also be regarded, not as bads, but rather as genuine goods. It is only in certain circumstances that these things, or the pursuit of them, becomes bad. He disagrees, therefore, with what he seems at times to regard as the view of Plato, that pleasure in itself is a bad thing, or “essentially a species of evil” (Aristotle, 1984a, VII, section 13, p. 1153b4–7, 1823).

In the *Nicomachean Ethics*, Aristotle argues that in order to be truly happy, each of us needs what he refers to as “the goods of the body” as well as “external goods,” or the goods “of fortune,” in order that we may avoid a life of pain and suffering (Aristotle, 1984a, VII, section 13, p. 1153b17–18, 1823). He also argues that a happy life, properly understood, necessarily requires a certain quantity of “external goods” (Aristotle, 1984a, I, section 8, p. 1099a32–1099b7, 1737; VII, section 13, p. 1153b22–24, 1823; X, section 8, 1178b33, 1863). For, he says, “our body also must be healthy and must have food and other attention” (Aristotle, 1984a, X, section 8, p. 1178b34–35, 1863). It is necessary therefore that human beings, in their pursuit of happiness, should pay at least some attention to what he regards as the “necessary pleasures,” or to their bodily needs. However, he again says that we should not go too far in this direction. For it is possible to pursue and to possess goods of this kind to “excess,” and in such cases they turn out to be an “impediment” to the successful pursuit of happiness, properly understood (Aristotle, 1984a, VII, section 13, p. 1153b22–24, 1823). The pursuit of goods of this kind should therefore be in moderation. Aristotle insists that being “sufficiently equipped with external goods” is at least a necessary condition for happiness, even if it is not a sufficient condition (Aristotle, 1984a, I, section 10, p. 1101a14–15, 1739). He says much the same in his other writings also, including the *Politics* (Aristotle, 1984c, VII, section 13, p. 1331b38-39-1332a1-2, 2113); the *Rhetoric* (Aristotle, 1984e, I, section 5, p. 1360b24-25, 2163); and his *Magna Moralia* (Aristotle, 1984b, II, section 8, p. 1206b33-34, 1909–1910).

To summarize the difference between the views of Plato and Aristotle regarding what is good, against what he takes to be the opinion of Plato, Aristotle insists that external goods, and indeed those goods which have to do with the human body also, really are genuine goods. They ought not to be thought of as bads, or things which are necessarily morally undesirable. Moreover, they can in principle make an authentic and significant contribution to human happiness, provided they are pursued in moderation and not to excess. Hence those who think in this way are not, as Plato argues, mistaken regarding what can or cannot properly be said to be a good. In the next section I compare what has been said above about the respective views of Plato and Aristotle regarding the different types of good with the views of Alasdair MacIntyre, especially but not only in *After Virtue*. I also assess the significance of what MacIntyre says about this issue for our understanding of his views regarding social institutions.

## Alasdair MacIntyre on “the corrupting power of institutions”

In *After Virtue* MacIntyre deploys the conceptual distinction between different types of good, in a somewhat different way from that of either Plato or Aristotle. Like Plato and Aristotle, he too employs the concept of “external goods.” Moreover, when talking about external goods he has in mind much the same things as they have in mind, for example such things as wealth, status and power. However, in his case, this idea is associated with issues having to do with sociology, specifically social institutions, rather than with individual psychology. There is a sense therefore in which MacIntyre might be said to personify social institutions by talking about what is good for them. The crucial question here is whether, when discussing social institutions and external goods, MacIntyre follows the lead of Aristotle or that of Plato. If he follows Aristotle, then he must think that external goods really are goods. If he follows Plato, then in his view external goods are not really goods at all, but bads, or things which ought to be avoided. I shall discuss these possibilities in turn.

### MacIntyre as an Aristotelian thinker

In *After Virtue* the disagreement between Plato and Aristotle regarding questions of psychology, about the nature of an individual person, and about what might be said to be good for them, has a sociological parallel in what MacIntyre says about social institutions and the relationship which exists between practices and institutions. There MacIntyre has a tendency at times to think of a social institution as if it is analogous to an individual person, as however understood by Aristotle rather than by Plato. MacIntyre is not entirely consistent when discussing this issue. Nevertheless, there are times when his commitment to a recognizably Aristotelian way of thinking about it is readily apparent.

MacIntyre is generally regarded as, and arguably also identifies himself as, a contemporary Aristotelian thinker, although in *After Virtue* he does not of course endorse everything that Aristotle has to say, for example his views on natural slavery, on women, or his metaphysical biology. Speaking generally, it might be said that whatever the issue that is debated between Plato and Aristotle, MacIntyre has a tendency to side with Aristotle against Plato. For example, in his *A Short History of Ethics*, he argues that, unlike Aristotle, Plato in the *Republic* “ignores,” with what MacIntyre regards as his characteristic and utterly deplorable puritanism,” the “many genuine bodily pleasures” which are identified by Aristotle (MacIntyre, 1998 [1967], p. 46). In the writings of Aristotle, MacIntyre argues, “there is nothing of Plato's at times nearly hysterical picture of what he takes to be the anarchy of desire” (MacIntyre, 1998 [1967], p. 98).

MacIntyre also says in the *Short History* that in Plato's writings the “mass of ordinary people” are presented “as governed by desires which have no room for expression in the just state.” Indeed, in Plato's *Republic*, again unlike in the writings of Aristotle, “the desires are to be repressed” (MacIntyre, 1998 [1967], p. 98). According to MacIntyre, “reason, in the Platonic scheme, can only dominate, not inform or guide, appetite, and appetite of itself is essentially irrational” (MacIntyre, 1998 [1967], p. 46–47). MacIntyre argues that this is a significant difference between the views of Plato and those of Aristotle. In the case of Plato, he claims, there is a “complete divorce of reason and desire in the soul.” This implies that for Plato, unlike for Aristotle, a “contrast has to be” drawn “between reason on the one hand and senseless and uncontrolled appetite on the other.” According to MacIntyre, “these are the only alternatives available, given the Platonic psychology” (MacIntyre, 1998 [1967], p. 47). MacIntyre contrasts this view with what he takes to be that of Aristotle, and of which he evidently approves, which is that “there is no *necessary* conflict between reason and desire, such as Plato envisages” (MacIntyre, 1998 [1967], p. 64).

We saw earlier that for Aristotle an individual person is regarded as a composite entity within which a particular mind or soul has been embodied. According to an Aristotelian reading of his views, MacIntyre says much the same thing about social institutions. He thinks that they too are composite entities, with just two component parts, which are in certain respects analogous to an individual human being. Consequently, in his opinion, a social institution can and should be thought of as being a particular “embodiment” of an underlying practice (MacIntyre, 1996, p. 290). In *After Virtue* MacIntyre says that a social institution, understood in this way, is necessarily the “bearer” of an underlying practice (MacIntyre, 2007 [1981], p. 222). He suggests there that, just like the mind or soul of an individual human being, so also a social practice could not exist at all unless it is embodied in some institutional form or other.

So far as the debate between Plato and Aristotle around the issue of the different types of goods is concerned, MacIntyre again tends at times to take the side of Aristotle against that of Plato. We have seen that for MacIntyre social institutions, as such, are necessarily oriented toward the pursuit of external goods. In MacIntyre's words, in *After Virtue*, “they are structured in terms of power and status, and they distribute money, power and status as rewards.” However, he continues, “nor could they do otherwise” if they “are to sustain not only themselves but also the practices of which they are the bearers” (MacIntyre, 2007 [1981], p. 194). MacIntyre insists, then, that practices cannot exist, or at least that “no practices can survive for any length of time,” if they are “unsustained by institutions.” Indeed, he argues, “the relationship of practices to institutions.” and consequently also that “of the goods external to the goods internal to the practices in question,” is “so intimate” that “institutions and practices characteristically form a single causal order” (MacIntyre, 2007 [1981], p. 194).

We have seen that according to one reading of his views, MacIntyre maintains that literally all social institutions are necessarily oriented toward the pursuit of external goods such as wealth, status and power. A critic of MacIntyre might, perhaps, cite the example of a monastery and ask whether the individual who occupies the role of an abbot in charge of a monastery might be regarded as a “manager” in MacIntyre's sense of the term. Nevertheless, it is a logical implication of what MacIntyre says about the relationship between practices and institutions that any individual who happened to occupy that particular social role would necessarily be involved to some extent in the pursuit of external goods.

The writings of St. Augustine are instructive here. I have in mind not only *The City of God* and his *Confessions*, but also his *The Rule of St. Augustine* and his *Of the Work of Monks* (St. Augustine, 1887, 1972, 1984a,b; see also Burns, 2020, 2021, p. 125–137). In *The City of God*, St. Augustine distinguishes between spiritual goods and temporal ones. On this issue, he appears to follow Aristotle rather than Plato, despite his reputation as a Platonist (Armstrong, 1966; O'Connell, 1984; Coleman, 1994). He characterizes temporal goods as being “extrinsic” goods, which are valuable as a means to an end, rather than for their own sake (St. Augustine, 1972, I, VIII, p. 8, 310; XIX, p. 17, 877). As examples, in his *Confessions*, he identifies the three “goods” of “honor, power and wealth” (St. Augustine, 1984b, II, p. 5, 48–49). In *The City of God* he refers to such things as “honor, glory, money, or the like” (St. Augustine, 1972, I, VIII, p. 8, 310). He also says that “it would be incorrect to say that the goods which this city desires are not goods, since even that city is better, in its own human way, by their possession” (St. Augustine, 1972, II, XV, p. 4, 599). In his *Of the Work of Monks*, Augustine discusses the management of the temporal affairs of the monastery of which he was a member, which necessarily involved paying at least some attention to the pursuit of these extrinsic goods. There he bemoans the fact that his administrative duties as an abbot, because of their association with the pursuit of these extrinsic goods, distracted him from his spiritual life as a Christian (St. Augustine, 1887, p. 37, 521).

Returning to MacIntyre, it is arguable that there is a straightforward analogy between what MacIntyre says about social institutions and what Aristotle says about individual persons. Aristotle holds that possession of external goods is a precondition for the achievement of those goods which he associates with the human body. However, in their turn, these “bodily goods” are a precondition for sustaining both the intellectual and the ethical life of an individual person, who necessarily also possesses a mind or a soul. For example, they make it possible for such agents to differentiate themselves from other species of animal by devoting themselves to what Aristotle regards as the higher pursuits of politics and philosophy. Similarly, MacIntyre argues that social institutions and their managers, together with the external goods which they necessarily pursue, are a precondition for sustaining the ethical life of a practice, or of a practice-based community, and of the practitioners who are its members.

Although he uses a different terminology, MacIntyre takes much the same view of the relationship which exists between practices and social institutions, and the types of “goods” with which they are associated, in *Whose Justice? Which Rationality?* There the equivalent of the earlier distinction between internal goods and external goods is the distinction which MacIntyre draws between “goods of excellence,” on the one hand, and “goods of effectiveness,” on the other. He suggests that the pursuit of these two different types of good “are to some large degree,” though (by implication) perhaps not necessarily, “linked together within the dominant social institutions” of a particular society. Indeed, he goes so far as to claim that this is true of all societies, everywhere, including that of ancient Greece at the time of Homer, long before that of Plato and Aristotle (MacIntyre, 1988, p. 32).

In *Whose Justice? Which Rationality?* MacIntyre maintains that “it would be a large misconception to suppose that allegiance to goods of the one kind necessarily excluded allegiance to the goods of the other” (MacIntyre, 1988, p. 35). The reason for this, he suggests, is because “those forms of activity within which alone it is possible to achieve the goods of excellence can only be sustained by being provided with institutionalized settings” (MacIntyre, 1988, p. 35). Moreover, “the maintenance of the relevant institutional and organizational forms,” which are the necessary embodiment of underlying practices, “always requires the acquisition and retention of some degree of power and some degree of wealth.” Again like Aristotle, when he talks about the necessary contribution to human happiness that is made by “external goods,” MacIntyre concludes that “the goods of excellence,” or the internal goods of practices, “cannot be systematically cultivated” by their practitioners “unless some at least of the goods of effectiveness,” or the external goods which MacIntyre associates with social institutions, “are also pursued,” presumably by their managers (MacIntyre, 1988, p. 35).

Perhaps the most significant reason for thinking that MacIntyre follows Aristotle rather than Plato when discussing the division of goods is the fact that he characterizes such things as wealth, status and power, not as being bads, or things which ought necessarily to be avoided, as Plato maintains at times, but rather as goods, or things which might legitimately be pursued. MacIntyre states explicitly in *After Virtue* that “I need to emphasize at this point that external goods genuinely are goods. Not only are they characteristic objects of human desire, whose allocation is what gives point to the virtues of justice and generosity, but no one can despise them altogether without a certain hypocrisy” (MacIntyre, 2007 [1981], p. 196). In making this remark, MacIntyre endorses the criticisms which Aristotle makes of Plato on this issue.

According to this Aristotelian reading of his views, MacIntyre holds with Aristotle and against Plato that things such as wealth, status and power are not bads, or things the pursuit of which ought to be avoided at all costs. Rather, he argues that although they are indeed goods, nevertheless they are of secondary importance when compared with the internal goods of practices. Moreover, just as in the case of individual persons, for social institutions and their managers the pursuit of these external goods is unavoidable. For they are the preconditions for the happiness or flourishing of a particular social institution, indeed for its very existence, just as the goods of the body are a pre-requisite for the health and wellbeing, or indeed the bare survival, of an individual human being or person. On this account, MacIntyre agrees with Aristotle that the pursuit of such external goods only becomes problematic, morally speaking, when it is carried out to excess. Provided it is moderate, this pursuit is morally permissible. Indeed, it is necessary.

Looked at in this Aristotelian way, precisely because a particular social institution is thought of as being the embodiment or the bearer of some underlying practice (MacIntyre, 2007 [1981], p. 222), it follows that it should not be regarded as being inherently bad or corrupt, even if it is in certain respects defective and there is a need for it to be reformed. This positive assessment of MacIntyre's views regarding the value of social institutions is reinforced by his remark that “the making and sustaining of forms of human community—and therefore of institutions—itself has all the characteristics of a practice” (MacIntyre, 2007 [1981], p. 194–195).

### Geoff Moore's Aristotelian reading of MacIntyre

This Aristotelian reading of MacIntyre may be associated with the writings of Geoff Moore (Moore, 2002, 2005a,b, 2008; Moore and Grandy, 2017). It can also be found in some (though not all) of the writings of Ron Beadle, especially those which he has jointly authored with Moore (Beadle and Moore, 2006, 2011; Moore and Beadle, 2006; Beadle, 2017). According to this reading, MacIntyre holds that just as practices and institutions should be regarded as being involved in a symbiotic relationship with one another (Beadle, 2008, p. 231; Beadle and Moore, 2011, p. 102), so too should practitioners and their managers. It would, therefore, be a mistake to assume that the relationship between these two groups of individuals is necessarily to be associated with any conflict of interests. Rather, provided that a particular institution is in a good state of sociological health (Emile Durkheim springs to mind at this point), their activities should be regarded as complementary or mutually supportive of one another.

This reading of MacIntyre assumes that, as in the case of wider society, so too there is a division of labor within each social institution, as a consequence of which each of these groups pursues the type of good with which it is characteristically associated, namely internal goods in the case of practitioners, external goods in that of managers. However, in so doing, each group is regarded, albeit in their different ways, as making a necessary contribution to the social life, health, wellbeing or “happiness,” and hence also to the common good, of the institution with which they are associated. It is only if the harmonious “balance” or “equilibrium” which ideally ought to exist between them is disturbed that a particular social institution might be regarded as having become for some reason morally corrupted (Moore, 2005b, p. 661, 678; Moore and Beadle, 2006, p. 376; Beadle, 2008, p. 231; Moore, 2008, p. 499, 503; Beadle and Moore, 2011, p. 100, 105; Moore, 2012a, p. 367; Moore, 2012b, p. 305, 310–311). An example of this would be if the managers of the institution in question were to choose to pursue external goods, not for the sake of the internal goods of the underlying practice, but rather for their own sake or to excess. In *Whose Justice? Which Rationality?* MacIntyre refers in this connection to the possibility that the managers of a particular social institution might systematically “subordinate goods of the one kind to goods of the other” (MacIntyre, 1988, p. 35).

According to this Aristotelian reading of MacIntyre, there might exist a corrupt social institution, in which the pursuit by managers of external goods does as a matter of fact obstruct or interfere with, to the point of actually preventing, the successful pursuit of internal goods by its practitioners. However, MacIntyre does not consider this to be inevitable. It is not something that is built into his understanding of the idea of a social institution as such. On this view, the idea of a “virtuous institution,” or a “virtuous organization,” or a “virtuous corporation,” or indeed a “virtuous manager,” makes perfectly good sense theoretically speaking (Moore, 2002, p. 28–30; Moore, 2005b, p. 663; Moore and Beadle, 2006, p. 374; Moore, 2008, p. 498–500; Beadle and Moore, 2011, p. 98–100). None of these ideas would, it is suggested, be regarded by MacIntyre as an oxymoron (Beabout, 2020, p. 210).

This conclusion rests upon an implicit argument, which runs as follows. If we have two social institutions, A and B, both of which are necessarily associated with the pursuit of external goods, to some extent at least, but one of which A is assumed to be a virtuous institution, whereas the other B is not, but, rather, a corrupt one, then it is evident that the difference between them, and the source of the corruption of B, cannot be the fact that B is an institution as such, or the mere fact that it necessarily pursues external goods. For that is true of A also. Rather, it must be the fact that in institution B external goods are pursued for their own sake or to excess, whereas in institution A they are not. Moore and Beadle have argued, on these grounds, that if he were consistent MacIntyre could not possibly argue that institutionalization in and of itself is the root cause of the ethical corruption of any practice. In other words, if a particular social institution has in fact become corrupted, this is not a problem of structure, having to do with the essential nature of all social institutions as such, but, rather, of agency, specifically that of the individuals who are responsible for the management of the institution in question (Beadle and Moore, 2006, p. 336; Moore and Beadle, 2006, p. 376; Beadle and Moore, 2011, p. 101; Moore, 2012a, p. 364–365, 380).

If we follow this argument through to its conclusion, then, given the initial Aristotelian assumption that external goods are genuine goods, and not bads, it follows that MacIntyre in *After Virtue* should not talk about “the corrupting power of institutions,” as if institutions in and of themselves are necessarily given over to corruption, or to the excessive pursuit of external goods for their own sake. Geoff Moore has rightly pointed out that a logical consequence of MacIntyre's insistence that external goods really are goods and not bads is that he must hold (or would if he were consistent) that “institutions are not, intrinsically, corrupt or corrupting of the practices” they embody (Moore, 2015, p. S104).

Indeed, it might even be argued that MacIntyre should not talk about the potentially corrupting power of institutions, or about an inherent tendency for them to corrupt. For it is an implications of this view, given the acceptance of the theoretical possibility that there are virtuous institutions, as well as virtuous managers, that such a corrupting power does not exist, not even potentially or as a tendency. On this view, there is no logical connection at all between the notion of a social institution and that of moral corruption. If there were, and institutions were thought of as being essentially or inherently corrupt or corrupting then, as Gregory R. Beabout has suggested, the expression “virtuous institution” would be a contradiction in terms (Beabout, 2020, p. 210). Given this, it is surprising that Moore and Beadle themselves seem willing at times to endorse MacIntyre's remark about the corrupting power of institutions (Moore, 2002, p. 22, 28, 30; Moore, 2005a, p. 250; Moore, 2005b, p. 661–663; Beadle and Moore, 2006, p. 332; Moore, 2008, p. 499; Moore and Grandy, 2017, p. 151).

### MacIntyre and Platonism?

It must be acknowledged that there is evidence which supports Moore and Beadle's Aristotelian reading of MacIntyre's views regarding external goods, and the relationship which exists between practices and institutions, that is presented in the preceding section. The broad thrust of that reading is that, at least some of the time, MacIntyre is not dismissive or entirely critical of social institutions and their managers. On the contrary, he simply calls for an ethical approach to the management of existing social institutions that is based on the assumptions of Aristotelian virtue ethics. The practical implications of this reading of MacIntyre's views are that although nothing radical is required, there may nevertheless be a need in certain cases for institutional reform.

However, an alternative reading is possible. For there are occasions when MacIntyre takes a very different view, especially of course when in *After Virtue* he refers explicitly to “the corrupting power of institutions” (MacIntyre, 2007 [1981], p. 194). The wording of this remark is categorical and unequivocal. MacIntyre does not say, as Moore and Beadle imply, that social institutions may be, or can be corrupting. Nor does he say that they are potentially corrupting, or that they have a tendency to corrupt. Rather, he suggests that they just are inherently corrupting and that they always do corrupt. His strongly worded assertion on this point implies that when making this remark MacIntyre assumed that social institutions as such are necessarily corrupting of the underlying practices with which they are associated. For this reason, they might be said to be bad, or deserving of our moral disapproval. On this occasion, then, MacIntyre suggests that the notion of corruption is “built in” to his understanding of the very concept of a social institution. Hence there are times when he does seem to assume that the idea of a “virtuous institution” or that of a “virtuous manager” is indeed an oxymoron.

According to MacIntyre, when he speaks in this way, it is a characteristic feature of all social institutions as such that within them external goods are necessarily pursued by their managers for their own sake or to excess. He associates this with corruption because he thinks that it is an obstruction lying in the way of the ethical life of its practitioners in their pursuit of internal goods. His remark about the corrupting power of institutions suggests that in his opinion the pursuit of external goods as a means to an end, namely the internal goods of a practice, and of doing so moderately, is a characteristic feature of practitioners and not of their managers. This implies of course that for MacIntyre practitioners can and do necessarily value external goods. They do not only value internal ones. So the difference between them and their managers is not and cannot be that they only value internal goods, whereas managers only value external ones. Rather, it is that practitioners value external goods as a means for achieving internal ones, whereas managers are assumed by MacIntyre, at least when arguing in this vein, to value external goods only for their own sake or to excess.

There is evidently a significant difference between making the recognizably Aristotelian claim that institutions as such are necessarily oriented toward the pursuit of at least *some* external goods, and making the far stronger claim that they are necessarily oriented toward the pursuit of external goods for their own sake or to excess. If one takes the former view, then there is nothing inherently or intrinsically bad or corrupting about social institutions. It is only if one takes the latter view that it makes sense to talk about institutions as such being corrupt or possessing a corrupting power. It might be argued on these grounds that when MacIntyre talks about the corrupting power of institutions in *After Virtue* he goes beyond what might be regarded as a straightforwardly Aristotelian position, i.e., one that simply acknowledges the need for an institution to pay at least some attention to the pursuit of external goods. If that were all he had in mind when making this assertion there would be no need for him to suggest that he regards institutions as essentially corrupting, or that they inherently possess a corrupting power.

This is a reason for thinking that there is an unacknowledged strain of Platonism in what MacIntyre says about social institutions when he talks about their corrupting power. Despite his stated intentions to the contrary, he does occasionally suggest that the pursuit of external goods is in itself a bad thing. As such, it is not merely potentially but actually corrupting. On this reading of his views, MacIntyre's assertion that social institutions are necessarily associated with moral corruption, implies that the pursuit of external goods is something which ought to be avoided, not simply because it makes the corruption of an institution possible, but rather because it leads inevitably to that result. When making this remark, MacIntyre goes beyond an Aristotelian position and comes close to a Platonic one.

There are a number of reasons for thinking that there is indeed a strain of Platonism in what MacIntyre has to say about external goods in *After Virtue*. One of these is that, like Plato, he has a tendency to associate the pursuit by individual agents of external goods with egotism, selfishness and greed. MacIntyre asserts at one point that external goods are typically “some individual's property and possession.” Moreover, “the more someone has of them, the less there is for other people.” They are therefore “characteristically objects of competition” between individuals, each of whom pursues their own self-interest as they understand it, rather than that of others, or the common good. This is a competition “in which there must be losers as well as winners” (MacIntyre, 2007 [1981], p. 190). It is for this reason that MacIntyre thinks that the pursuit of external goods in and of itself is corrosive of the ethical life of a community of practitioners in their joint pursuit of a common good.

A second reason, or perhaps the same reason restated in a different way, is that MacIntyre has a tendency to associate the pursuit of external goods with vice rather than with virtue. We saw earlier that MacIntyre defines the concept of virtue in such a way that it is necessarily associated with “those goods which are internal to practices” (MacIntyre, 2007 [1981], p. 191). However, he also maintains that it is “the lack of” virtue which “prevents us from achieving any such goods” (MacIntyre, 2007 [1981], p. 191). This might be thought to imply that when the achievement by practitioners of the internal goods of a practice is prevented, this is because of the presence of vice. However, MacIntyre seems at times to assume that practitioners as such are necessarily virtuous. If therefore their efforts to achieve the internal goods of a practice are obstructed, the presumption is that this occurs because of the vicious actions of the managers of the social institution which embodies that practice.

Like both Plato and Aristotle, then, MacIntyre sometimes associates the pursuit of external goods, and therefore also social institutions and their managers, not with virtue, but rather with vice, specifically the vice of “acquisitiveness” or greed (*pleonexia*). This is the demand not merely for some or enough external goods, but for more of such goods than is necessary. In *After Virtue* MacIntyre says that the moral “ideals” of a particular practice “are always vulnerable to the acquisitiveness of the institution” by which it is embodied (MacIntyre, 2007 [1981], p. 194). He assumes therefore that institutions as such are essentially acquisitive, that is to say, vicious. This is why MacIntyre maintains there, albeit somewhat cautiously, that whereas on the one hand “the integrity of a practice” requires “the exercise of the virtues by at least some of the individuals who embody it in their activities,” on the other hand the “corruption of institutions” is “always in part at least an effect of the vices” (MacIntyre, 2007 [1981], p. 195).

I have characterized this reading of MacIntyre's views as Platonic. The main reason for doing so is that, on this reading of MacIntyre's views, just as Plato has a “down” on the body and on all alleged goods which are associated with it, which in his opinion are not really goods at all but bads, so too MacIntyre seems when talking about “the corrupting power of institutions” to have a similar “down” on social institutions and on their pursuit of external goods, which he regards as being essentially vicious, because they are acquisitive. When making his remark about the corrupting power of institutions, MacIntyre implies that in his view it is not possible for a social institution or its managers to pursue external goods in moderation. Rather, he seems to assume that it is inevitable that if they pursue them at all then they will be driven to pursue them acquisitively, for their own sake and to excess.

According to this Platonic reading of his views, when making his remark about the corrupting power of social institutions, MacIntyre assumes like Plato that external goods are not goods at all. Rather, they are bads, or things which ought to be avoided. As such they are not merely sources of possible corruption, because they have a potential or a latent tendency to lead to excess. Rather, they are sources of actual corruption, because the drive to acquire external goods to excess is somehow built in to them. Hence, to pursue these goods at all, to any degree, is corrosive of ethical life. Those who wish to live an ethical life, a life of justice, co-operation and mutual fellowship with others in a practice-based community, ought therefore to avoid their pursuit.

The wording of MacIntyre's claim that social institutions as such possess a corrupting power implies that he thinks that there is and can be no harmony of interests between practitioners and managers in any social institution. Rather, there is necessarily a conflict of interests between them in their respective pursuits of the different types of good with which they are associated. Nor, therefore, is it the case that each of these two groups can be thought of as making, in their different ways, a contribution to the common good of the social institution with which they are associated. For it is practitioners who are motivated by the virtues to do this, whereas managers are not. The managers of social institutions are not merely potentially but actually given over to vice. They inevitably pursue external goods for their own sake or to excess. When making his controversial remark, MacIntyre implies that he thinks that this is their *raison-d'etre*.

MacIntyre's remark in *After Virtue* about the corrupting power of institutions assumes that social institutions as such are necessarily associated with vice rather than with virtue, and hence also with the excessive pursuit of external goods at the expense of internal ones. From this point of view, it follows that the notion of a virtuous institution or a virtuous manager is a logical impossibility. It is indeed an oxymoron. Hence, of course, no such thing could possibly exist as a matter of empirical fact. From the standpoint of a Platonic reading of MacIntyre, this possibility is automatically ruled out of court by definitional fiat, as a consequence of the way in which the meaning of the relevant theoretical concepts are understood.

Not surprisingly, Moore and Beadle have taken issue with what they take to be the *a prioristic* or axiomatic approach to this issue which MacIntyre seemed to adopt when making his remark. They suggest that MacIntyre's theory, as he sometimes formulates it, has two defects. First, it is a blunt-instrument, because it is formulated at too high a level of generality. Second, it is not empirically falsifiable, as (perhaps following Sir Karl Popper) they assume all good explanatory theories should be. Moore and Beadle cite Traidcraft plc. as an empirical example of what they consider to be a “virtuous corporation” (Moore and Beadle, 2006, p. 381–384). Despite MacIntyre's suggestion that this is not possible, they regard this example as providing empirical evidence which counts against his theory. Hence, they argue, the theory needs to be reformulated.

MacIntyre evidently does not want to associate himself too closely with Plato and Platonism, as opposed to Aristotle and Aristotelianism, and yet his assertion about the corrupting power of institutions is arguably more Platonic than Aristotelian in terms of the underlying assumptions upon which it rests. It makes just as little sense to say that institutions have a corrupting power as it does to say that the human body has a corrupting power. This is true only in the trivial sense that if human beings did not have bodies, with associated desires, then their ethical corruption as Aristotle understands that idea could not possibly occur. The question here is, given that all human beings do necessarily possess bodies, why is it that some of them are in fact virtuous whereas others are not? Aristotle does not accept what he takes to be Plato's view that it is the body in itself which is the source of the problem of moral corruption, because it is necessarily associated with vice, or because it necessarily makes people vicious. He rejects the idea of the inherent corruption, and hence also of the corrupting power, of the human body.

Similarly, in the case of social institutions, the parallel issue, as Geoff Moore and Ron Beadle have observed, is why one social institution or business organization is virtuous whereas another is not. If the analogy between the two cases is drawn, then we would have to conclude that a consistent Aristotelian would not accept that it is institutionalization as such, or the institutional embodiment of a practice, that is, or ever could be, the source of the problem, given MacIntyre's acknowledgment that all practices need to be embodied in some institutional form or other. However, in that case of course it follows that it makes just as little sense for a supposedly Aristotelian thinker to talk about the corrupting power of social institutions as it does to talk about the corrupting power of the human body.

## Conclusion

MacIntyre's remark in *After Virtue* about the corrupting power of institutions as such may be associated with a radical critique of contemporary social institutions in the advanced, industrial (or post-industrial), bureaucratised, capitalist societies of the global North and West. However, in order to speak in this way about social institutions it appears, at first sight anyway, that he must embrace a Platonist and not an Aristotelian way of thinking about external goods. Given this, it is not too surprising that Geoff Moore has suggested that MacIntyre “is his own worst enemy” (Moore, 2002, p. 19). That is to say, he was not being true to his own Aristotelian assumptions when he made this remark. Moore and Beadle have cited with apparent approval the opinion of some of the contributors to a volume discussing whether or not education is a practice, who “go almost so far as to assert that” there are occasions when MacIntyre appears not to “understand his own concept” (Dunne and Hogan, 2004; Beadle and Moore, 2006, p. 334–335). A similar view might perhaps also be taken in connection with MacIntyre's remarks about institutions.

MacIntyre's statement about the corrupting power of institutions suggests that there is an inconsistency, if not in what he thinks then at least in what he says at times about social institutions and their pursuit of external goods. This raises some interesting methodological and ethical issues relating to the reading of texts (Burns, 2011a,b). One of these is how much significance should be attached to the stated intentions of an author? Another has to do with what is to be done when, as here, an author's remarks are ambiguous, or he appears to contradict himself? In such cases, there is a need to “reconstruct” the thought of the author in question, something which can always be done in more than one way. There is, therefore, the further question of how this act of reconstruction is to be carried out? How exactly should these ambiguities, or these real or apparent inconsistencies be resolved?

In the case of MacIntyre on practices and institutions, one way of dealing with this issue would be to talk only about the possible rather than the actual or inevitable corruption of practices, not by the social institutions which embody them as such, but rather by their individual managers. In that case, as Geoff Moore has argued, if and when corruption does in fact occur, this has to do with the choices which are made by those who happen to occupy its managerial positions. As we saw earlier, Moore and Beadle maintain that corruption, within institutions, whenever and wherever it does in fact occur, is not a structural issue. It is solely a matter of individual responsibility or moral agency.

A second way of resolving this problem of apparent inconsistency would be to lower the level of abstraction at which MacIntyre's critical remarks about social institutions are expressed and fine-tune his theoretical approach by drawing a distinction between different types of social institution and talking about the corrupting power, not of all institutions, but rather of only some of them. For example, the size and level of bureaucratization of a particular institution might be regarded as significant here, or the fact that it is located within a pre-modern society, a modern one, or in a capitalist as opposed to a non-capitalist one. As we saw earlier, Moore and Beadle have drawn attention to the fact that MacIntyre's occasional blanket denunciation of all social institutions as such makes it impossible for him or us to acknowledge the existence of any virtuous institution, or any virtuous business corporation, for example Traidcraft plc.

Samantha Coe and Ron Beadle have drawn attention to the fact that MacIntyre appears at times “to distinguish between different types of institution,” considered “in respect of their relationship to practice” (Coe and Beadle, 2008, p. 9). They indicate that although MacIntyre does state explicitly that a practice cannot exist (at least not for long) without being embodied in an institution, he seems to have accepted that it is possible for an institution to exist without being associated with any underlying practice. There are occasions when he differentiates between practice-embodying and non-practice-embodying social institutions (MacIntyre, 1996, p. 290). If so, then of course institutions of the latter type would necessarily be focused exclusively on the pursuit of external goods such as wealth, status and power. Such an institution would be entirely “corrupt,” morally speaking, irrespective of whether one is talking about the Platonic or the Aristotelian understanding of that term. If it is assumed that the institution in question had at some point previously been a practice-embodying one, then it seems appropriate in this context to talk about the “colonization” of practices by social institutions, in the sense in which Jurgen Habermas understands that concept (Habermas, 1984 [1981], p. 196, 305, 391–395). Kelvin Knight has rightly suggested that there is an affinity between MacIntyre's distinction between practices and institutions and Habermas's distinction between system and lifeworld (Knight, 1998, p. 293; but see also Breen, 2012, p. 186).

Because of the ambiguity and inconsistency of the wording which he employs at times, MacIntyre's views regarding social institutions and those who manage them can be and have been understood in very different, indeed diametrically opposed ways. As we have seen, he might be and has been read as an advocate of institutional reform, who relies heavily on the ideas of Aristotle, especially Aristotle's view that external goods really are genuine goods. Alternatively, he might be and has been read as a radical critic of existing social institutions and of those who manage them, who implicitly assumes as Plato also did that these external goods are not genuine goods at all, but are rather intrinsically bads, which as such ought to be avoided. We do seem therefore, at least at first sight, to be compelled to make an either-or choice between understanding MacIntyre as an Aristotelian thinker but also an institutional reformer, on the one hand, and understanding him as a radical critic of social institutions, but also as a latter day Platonist on the other.

There are some commentators at least who find neither of these alternative readings of MacIntyre congenial. In their view, this disjunctive approach overlooks the possibility that MacIntyre can and should be regarded as an Aristotelian thinker and not a Platonist, for obvious reasons, but one who at the same time is to be associated with a radical critique of contemporary society and its institutions. He is, in short, a “revolutionary Aristotelian” thinker and not a reformist one. Those who read MacIntyre in this way tend to attach importance to his relationship with Marxism, as well as his commitment to Aristotelianism. In their view, the fact that the institutions which are criticized by him are predominantly capitalist, or situated within a capitalist society, is of paramount importance. This third possible reading has received some discussion in the literature on MacIntyre (Blackledge and Davidson, 2008; Blackledge and Knight, 2011). Although I am broadly sympathetic toward this reading, I have suggested elsewhere that there are occasions at least when MacIntyre seems to be as much concerned about bureaucracy as he is about capitalism, and that his understanding of modernity appears to owe as much to the writings of Max Weber as it does to those of Karl Marx (Burns, 2011c; see also Burns, 2005). Discussion of how it is possible for MacIntyre to be an Aristotelian thinker whilst also being a radical critic of existing social institutions is connected to a discussion of the attitude of both Aristotle and MacIntyre toward utopianism, especially in its Platonic form (see Burns, 2019). However, that is a subject requiring an extensive treatment, which will have to be left for another occasion.

## Data availability statement

The original contributions presented in the study are included in the article/supplementary material, further inquiries can be directed to the corresponding author/s.

## Author contributions

The author confirms being the sole contributor of this work and has approved it for publication.

## Conflict of interest

The author declares that the research was conducted in the absence of any commercial or financial relationships that could be construed as a potential conflict of interest.

## Publisher's note

All claims expressed in this article are solely those of the authors and do not necessarily represent those of their affiliated organizations, or those of the publisher, the editors and the reviewers. Any product that may be evaluated in this article, or claim that may be made by its manufacturer, is not guaranteed or endorsed by the publisher.
